# Nano-Encapsulation of Citrus Essential Oils: Methods and Applications of Interest for the Food Sector

**DOI:** 10.3390/polym14214505

**Published:** 2022-10-25

**Authors:** Ioana Oprea, Anca Corina Fărcaș, Loredana Florina Leopold, Zoriţa Diaconeasa, Cristina Coman, Sonia Ancuța Socaci

**Affiliations:** Faculty of Food Science and Technology, University of Agricultural Sciences and Veterinary Medicine, 3–5 Calea Mănăștur, 400372 Cluj-Napoca, Romania

**Keywords:** citrus essential oils, nano-encapsulation, nano-emulsions, nanoliposomes, polymers, antimicrobial, antioxidant

## Abstract

Citrus essential oils possess many health-promoting benefits and properties of high interest in the food and agri-food sector. However, their large-scale application is limited by their sensitivity to environmental factors. Nanostructures containing citrus essential oils have been developed to overcome the high volatility and instability of essential oils with respect to temperature, pH, UV light, etc. Nanostructures could provide protection for essential oils and enhancement of their bioavailability and biocompatibility, as well as their biological properties. Nano-encapsulation is a promising method. The present review is mainly focused on methods developed so far for the nano-encapsulation of citrus essential oils, with emphasis on lipid-based (including liposomes, solid lipid nanoparticles, nanostructured lipid particles, and nano- and micro-emulsions) and polymer-based nanostructures. The physico-chemical characteristics of the obtained structures, as well as promising properties reported, with relevance for the food sector are also discussed.

## 1. Introduction

Essential oils (EOs) are secondary metabolites of plants. They are highly volatile and limpid liquids, generally soluble in organic solvents [1,2]. According to the International Organization of Standardization, EOs are defined as “the product obtained from vegetable raw material, either by distillation with water or steam, or from the epicarp of citrus fruits by a mechanical process, or by dry distillation” (ISO 9235, 1997). EOs had been discovered in the 16th century and gained their name from the drug *Qinta essentia*, also called essences due to their flammability [2]. The chemical composition of EOs varies greatly depending on intrinsic (e.g., botanical origin) and extrinsic (e.g., geographical origin) factors. From the chemical point of view, the composition of EOs includes terpenes and terpenoids, phenylpropanoids, and short-chain aliphatic derivatives, characterized by low molecular weight. Of these, the major ones are the mono- and sesquiterpenoids including alcohols, aldehydes, ketones, phenols, oxides, etc. Both the major and minor constituents are contributing to the bio-functional properties (e.g., antioxidant activity, bacteriostatic activity, antimycotic activity) and to the aroma of the EOs [1,3]. There are EOs containing only a few chemical compounds but also EOs that are complex mixtures of tens or even more than 100 compounds [1].

Citrus EOs are one of the most common EOs being extracted from the peel of citrus fruits. Citrus EOs have different compositions linked to the geographic location and harvest stage of the fruits, but their main shared component is the monoterpene limonene, found in proportions between 32 and 98%. Limonene is considered both flavoring and fragrant agent [4,5]. Other compounds identified in the citrus EOs are α and β-pinene, β-myrcene, sabinene, γ-terpinene, α-terpinolene, verbenone, linalool, citronellal, α-terpineol, geraniol, terpinen-4-ol, nerol, limonene oxide, etc., as flavoring agents but also compounds considered fragrances such as β-phellandrene, Δ-Carene, (E)-β-ocimene, linalool acetate, citronellal acetate, etc. [6,7].

Citrus EOs are known to have strong antioxidant, antifungal, antibacterial and insecticidal properties with manyfold applications in the food industry, pharmaceuticals, sanitary, and cosmetics [4]. They are recognized for various health-promoting benefits such as antiallergic and anti-inflammatory properties, anticancer activities, hepatoprotective and hypoglycemic effects, sedative, and antidepressant-like effects, used as an alternant treatment in inflammatory skin diseases and respiratory tract infections, protective effect on acute liver and kidney damage. However, their most widely studied properties are those related to their antioxidant and antibacterial activity [6].

For example, the tangerine EO potential in the prevention of atherosclerosis has been proved by Castro et al. through inhibition of lipid synthesis and a decrease in LDL lipid peroxidation [8]. In addition to the hypolipidemic properties, tangerine EO also presents anti-inflammatory properties and it is widely used for its antidepressant effects [9]. Orange EO is recognized for a broad list of health-promoting benefits such as anti-obesity effect through reduction of total cholesterol by 17% [10], antidepressant and anxiolytic [11], apoptotic and anti-angiogenesis effects on colon cancer cells [12], anti-inflammatory, neuroprotective, analgesic effects field [13], etc. Bergamot EO is well recognized in the literature for its anticancer properties and its suitability in food preservation [14].

Citrus EOs can exhibit good antimicrobial properties on strains such as *Listeria monocytogenes*, *Salmonella* spp., *Escherichia coli*, *Staphylococcus* spp., *Saccharomices* spp., *Aspergillus niger*, *Pseudomonas aeruginosa*, and *Xanthomonas* spp. [7,15,16], for this reason, they are intensely approached in the food preservation industry and research.

Their applications in food preservation are nowadays best illustrated by citrus EO-loaded biofilms used mostly with the purpose of shelf life extension of different food products such as fish [17], or any other food products [18]. The necessity of transition from synthetic food additives to natural ones qualified citrus EO as a possible natural additive in the food industry [19].

Citrus EOs are frequently studied for their antimicrobial and antioxidant activity but their main use is food industry remains as flavoring agents [20]. Their flavor is also based on the terpenoid compounds and due to their instability food industry shifted from the use of natural extracts to the use of similar synthetic compounds. Nano-encapsulation of citrus EOs can be a solution for the use of natural flavoring instead of synthetic compounds [21].

Even though citrus EOs have promising potential, with many health-promoting benefits and applications in the food industry, their susceptibility to external factors such as temperature changes, UV radiations, and pH changes limit their applications in the food industry or other sectors. Protection of bioactive compounds and enhancement of their bioavailability and biocompatibility is a primary concern in scientific research and encapsulation techniques are highly studied and perfectioned, especially nano-encapsulation technics and technologies, on the grounds that nanostructured compounds provide a series of advantages in modern applications of the food industry (e.g., increase in surface:volume ratio, increase in stability, increase in cellular permeability, increase in antibacterial and antioxidant potential, controlled release of bioactive compounds) [22]. There are multiple options regarding the encapsulation of EOs into nanometric-sized particles which include polymer-based nanocarriers, lipid-based nanocarriers, and molecular complexes [3].

Based on their chemical origin, the nanostructured systems suitable for EOs delivery are categorized into two large groups. The following discussion will be focused on: (i) lipid-based nanostructures comprising liposomes, nanoemulsions, solid lipid nanoparticles, and self-nano-emulsifying drug delivery systems [23] and (ii) polymer-based nanostructures including nanocapsules and nanogels, with emphasis on the synthesis methodology, on the physical–chemical properties of the nanostructures, stability, and on their relevant properties for the food sector.

## 2. Lipid-Based Nanostructures as Carriers for Citrus Essential Oils

### 2.1. Nano-Emulsions

The literature data show that nano-emulsions have the widest interest in the nano-encapsulation of citrus EOs into lipid-based nanostructures.

Emulsions are defined as the dispersion of two immiscible liquids in which the dispersed phase is found in the form of droplets in the continuous phase [24,25]. Water and oil are the most widely used components in an emulsion along with a substance (emulsifier) meant to stabilize the dispersion by reducing the interfacial tension formed between the dispersed phases [24,26]. Based on the proportion of aqueous and lipid phases, several types of emulsions can be distinguished, such as oil in water emulsions (O/W), water in oil emulsions (W/O), and complex emulsions systems such as water in oil in water emulsions (W/O/W) or oil in water in oil emulsions (O/W/O) [24].

Based on the size of the dispersed droplets macro-emulsions, micro-emulsions and nano-emulsions can be distinguished. Macro-emulsions are kinetically stable and thermodynamically metastable structures with droplet sizes in the range of 1 and 100 µm and optically turbid. They have high polydispersity and they are characterized as temperature and pH stable [24].

The ground rules of macro-emulsions are also valid for both micro-emulsions and nano-emulsions, but their input consists of higher membrane permeability and different stability in time. According to Anton and Vandamme [27] and many others, nano-emulsions (NEs) can be defined as kinetically stable systems, but they differ from micro-emulsions in terms of thermodynamical stability, the last ones being the stable ones [26,27]. For NEs the size of droplets generally varies from 20 to 500 nm [28,29]. The common aspects of NEs and micro-emulsions are the similar low polydispersity but the advantage of NEs in comparison with micro-emulsions is their stability in changes in temperature and pH [24]. Even though there are similarities between micro-emulsions and nano-emulsions, the main difference consists in an equilibrium between all components in the case of micro-emulsions that allows them to separate in different phases at any time. This is not the case for nano-emulsions, which show much longer stability over time [30].

NEs possess great potential in different fields, and they gained high popularity in pharmaceutics, biotechnologies, and the food industry, due to their capacity to increase the bioavailability of lipophilic compounds by increased interfacial areas. Coalescing, sedimentation and flocculation are prevented for long periods of time by the small dimensions of droplets and the Brownian motion present which give them the title of kinetics stable particles although they are considered disequilibrated systems [29]. Considering the above-mentioned aspects, NEs are more suitable as carriers for citrus EOs due to their unchanged physical properties over long periods of time.

NEs can have different forms such as oil in water (O/W) or water in oil (W/O) depending on the proportion of hydrophobic and hydrophilic compounds [31]. Water in oil NEs are represented by hydrophilic droplets dispersed into an oleaginous continuous phase. They can incorporate hydrophilic components and act as carriers for them. Oil in water NEs consist of a lipophilic core able to incorporate lipophilic components dispersed into a continuous aqueous phase. Due to the hydrophobic character of EOs, the present paper will focus on the discussion on oil in water NEs.

NEs can be obtained by two different classes of methods based on the energy level consumed in the process. These are high-energy techniques (HE) (e.g., high-pressure homogenization) and low-energy techniques (LE) (e.g., phase inversion temperature). LE techniques are less recognized as NEs generators as a result of frequent confusion in the workflow that can easily generate micro-emulsions instead of NEs [26]. NEs obtained by HE processes are based on intense levels of energy applied on macroemulsions capable to reduce the size of the dispersed droplets at the nanometric scale [32,33,34]. The main advantages of HE processes consist of reproducible results regarding the size of droplets and polydispersity index (PDI) and also the potential to scale the process at the industrial level [31]. HE techniques can be classified according to the devices used in the process of high-shear homogenization (HSH), high-pressure homogenization (HPH), high-pressure microfluidization (HPM), and ultrasonic homogenization (UH).

#### 2.1.1. High-Energy Techniques for the Obtaining of Citrus Essential Oils NEs

HPH has been reported in the literature as a means to obtain NEs with D-limonene, a major component of citrus essential oil, with droplet size ranging between 130 and 360 nm and PDI values from 0.07 up to 0.18, depending on the emulsifier used and the compound ratio into the oil phase. Donsi et al. compared the difference in the size of droplets and polydispersity for natural (soy lecithin, pea proteins) and synthetic (sugar esters, glycerol monooleate, and Tween 20) emulsifiers, and the results obtained were similar, even better for natural emulsifiers namely pea proteins. The NEs also had antibacterial activity against *S. cerevisiae*, *E. coli*, *L. delbrueckii* sp. *Lactis*, except for those obtained based on pea proteins [35,36]. The first article compared the minimum inhibitory concentration for four types of D-limonene NEs obtained by the HPH method using different types of surfactants and different lipidic substrates on the three microorganisms species mentioned above and they reported enhanced antimicrobial properties of D-limonene loaded nanostructures compared to pure D-limonene (>25 g/L for D-limonene for all three species, 10 g/L for D-limonene NEs with clear gum and soy lecithin as surfactants and 5 g/L for D-limonene and a mix of Tween 20 and glycerol monooleate as surfactant for all three species of microorganisms) [35]. The second study determined logarithmic inhibition of the same three species of microorganisms in 24 h for different NEs loaded with D-limonene also obtained by the HPH method. The study reported total inhibition of all four NE types in the case of *S. cerevisiae* after 24 h and also the NEs with a blend of glycerol monooleate and Tween 20 as surfactants exhibited total inhibition of *L. delbrueckii* and *E. coli* strains. The less effective NEs had pea proteins as surfactant agents and they did not exhibit any inhibition for *L. delbrueckii* and *E. coli* [36]. Additionally, HPH was reported as a method in the production of bergamot EO NEs using as surfactants the whey protein isolate, sugar esters, or glycerol monooleate. NEs were obtained by the same technology but with different surfactants and different bergamot EO concentrations. The resulting NEs presented high fluctuations in size and PDI (e.g., from an average of 73 nm in diameter and a PDI of 0.16 for NEs obtained with 0.07% bergamot EO and glycerol monooleate combined with Tween 20 as surfactant up to 298 nm and a PDI of 0.29 for NEs obtained with a mixture of whey protein isolate and modified starch as surfactant and 5% bergamot EO). The NEs had antibacterial activity (*E. coli*, *L. delbrueckii*, *S. cerevisiae*), as well as cytotoxic properties on Caco-2 cells, properties which were enhanced for the nanostructures compared to free oils [14].

UH has been used to obtain NEs from orange peel EO with droplet sizes between 21 and 95 nm with a focus on optimization of the nanostructure synthesis process [37,38]. UH obtained NEs based on tangerine, grapefruit, and lemon EOs [39] were also reported. In all cases, Tween 80 was used as an emulsifier due to its GRAS status, in concentrations of 1 and 2% (*v*:*v*). Yagzan et al. focused on comparing the antibacterial effect of lemon EO and lemon EO-loaded NEs on common fish spoilage bacteria (i.e., *P. damselae*, *E. fecalis*, *V. vulnificus*, *P. mirabilis*, *S. liquefaciens* and *P. luteola*) and food-borne pathogens (i.e., *S. aureus*, *K. pneumoniae*, and *S. paratyphi A*) and concluded that nano-emulsification increased the antibacterial effect of lemon EO for most of the studied bacteria. The study assessed the inhibition zone of lemon EO NEs in comparison with the same concentration of pure EO as in the loaded NEs and obtained higher inhibition zones for most food-borne pathogens (for pure EO/loaded NEs the inhibition zone were 16.25/16.63 mm for *S. aureus*, 10.5/24.25 mm for *E. Fecalis*, 11.50/14.38 mm for *K. Pneumoniae* and the only reverse example is for *S. paeatyphy* with 24.25/17.88 mm) [39].

Guerra-Rosas et al. used the HPM method in pursuance of obtaining tangerine-loaded nanostructures and studied their stability over time and the antimicrobial activity on typical food-borne pathogens (i.e., *E. coli* and *L. innocua*). The study attested to the achievement of NEs with an average droplet size of 17 nm and presented higher time stability over other EOs (i.e., oregano and thyme EOs) [40].

#### 2.1.2. Low-Energy Techniques for the Obtaining Citrus Essential Oil NEs

LE methods have not always been recognized as being suitable for obtaining NEs [30] due to possible confusion in the process of obtaining that could easily lead to micro-emulsions if the order of reagents is not respected strictly [26]. Low-energy methods for NEs production are spontaneous methods that require a minimum amount of energy and rely on physical-rheological properties of the mixture (e.g., interfacial tension). The two most used low-energy techniques are the emulsion inversion point (EIP) and phase inversion temperature (PIT) [41].

EIP is also known in the literature as phase inversion composition or spontaneous emulsification [42]. Its main restriction in forming nano-emulsions instead of micro-emulsions consists of the mixture of the emulsifier directly with the lipophilic phase before the addition of the aqueous phase. In this process, macroemulsions are formed first as W/O emulsions and therefore the aqueous phase is added slowly in order to catch the inversion point where interfacial tension of the O/W interface has a low value that allows the formation of nanometric-scale droplets as O/W NEs [41]. A great interest in the use of the EPI method to form nano-emulsions of citrus EOs, such as lime EO [43] and finger citron EO has been exhibited [44]. The studies presented droplet sizes below 100 nm and fine stability over time, from 4 up to 12 weeks. The emulsifier used in the experiments was either chitosan or Tween 80 both with high biocompatibility and accepted as GRAS. EPI was used in the case of D-limonene with polyoxymethylene as an emulsifier with droplets sizes in the range of 112–594 nm depending on the concentration of the surfactant [45].

Additionally, a combination of low and high energy methods has been reported in order to obtain orange EO NEs [46] with droplets under 100 nm and PDI between 0.2 and 0.4. In this experiment Tween 80 was used as emulsifier and the focus of the study was on the influence of the oil phase concentration and the emulsifier concentration to the droplet size and polydispersity index of the NEs. As LE methods it was used EPI and for the HE methods ultrasonication was performed.

The NEs obtained from citrus EOs and citrus EOs components were reported to have better antimicrobial properties compared to pure EOs/EOs components for species such as *E. coli* [47], *L. delbrueckii*, *S cerevisiae* [35,36], *B. subtilis* [44], *Salmonella* spp. and *S. aureus* [43].

Liew et al. studied the antimicrobial effect of NEs obtained by EPI method loaded with three lime species EO for *E. coli*, *Salmonella* spp., and *S. aureus* and he obtained inhibition zones between 7 and 10 mm for all species on fresh NEs and values between 6 and 8 mm after 1 month of storage of the loaded NEs [43]. Li et al. studied the minimum inhibitory concentration of finger citron EO loaded NEs obtained by spontaneous emulsification with different types of emulsifiers in comparison with pure EO and they obtained higher antimicrobial activity for NEs obtained with Tween 20 in case of *B. subtilis* and *E. coli* (0.16 mg/mL but also for the NEs obtained with Tween 80 for *S. aureus* (0.16 mg·mL) [44].

In the case of *E. coli*, the survival rate of the bacteria in vitro was compared upon exposure to three different inhibitors. For the first experiment 52 °C treatment was applied for 1 h. The second experiment consisted of treatment of *E. coli* with a suspension of *Citrus sinensis* oil and the third experiment consisted of treatment of bacterial cultures with chitosan nano-emulsions containing *Citrus sinensis* oil. A combination treatment with citrus oil/nano-emulsions and heat was also applied. The authors compared the results obtained by combined exposure to nano-emulsions and heat treatment, with the exposure of heat treatment alone and an increase in inhibition power up to five times in only 25 min was observed [47].

In addition to antibacterial effects, the nano-emulsions obtained with citrus EOs also showed great antioxidant properties for unsaturated fatty acids, qualities that recommend this carrier system for use in the food industry, mainly in food preservation [48].

Table 1 summarizes the types of methods and emulsifiers used for different EOs NEs and also the results regarding the droplet sizes and PDI. The literature reports heterogenous data for the particle size, even for the same EO, in accordance with the technique and emulsifier used in the process. As revealed by the data presented, high-energy techniques have more popularity compared to low-energy techniques.

### 2.2. Liposomes

Liposomes can be defined as vesicles formed by two layers of phospholipids that are self-assembled and sealed to the aqueous environment in which it is found. Such structures have both hydrophobic and hydrophilic character as a result of the phospholipid properties [50,51,52]. They can be formed from cholesterol and other biocompatible phospholipids of natural origin and can range in size from 10 nm up to micrometers [50,51]. The two major components of liposomes are the hydrophilic head consisting of phosphoric acid linked to a hydrophilic molecule and the hydrophobic tails composed of two fatty acid chains with 10–24 carbon atoms and up to 6 double bonds [50]. The most commonly used phospholipids in liposomal production are phosphatidylcholine, a natural compound also known as lecithin, and also phosphatidylethanolamine and phosphatidylserine [50].

Liposomes began to be considered as nanocarriers in the 1990s. They are considered to be highly versatile in properties such as shape and size and can be divided into unilamellar liposomes, oligolamellar liposomes, and multilamellar liposome [53]. Multilamellar are the most common used types of liposomes due to their increased stability over time and their ability of controlled release of the cargo in a higher timeline [54].

Liposomes are currently used in delivery of various types of EOs including citrus, in order to improve the antioxidant and antimicrobial properties of the oils and also increase their stability [55,56,57]. Since EOs are hydrophobic, they can easily be included in the phospholipidic layers of liposomes [58].

Liposomes’ properties are dependent on their synthesis processes, such as dehydration and rehydration, reverse phase evaporation vesicles prepared by extrusion, and frozen and thawed methods [53]. Each synthesis process requires different types and levels of energy in order to induce the formation of liposomes, a fact that is highly important in the final size and properties of the particles. The phospholipids used in the process also have a crucial role [54].

Another important factor for liposomal nanocarriers is the loading of active components. This process affects the liposome properties, as well as their loading efficiency. Loading can be active or passive loading. Active loading involves pH changes and differences in electrical potential and has proven to be more effective that the passive loading [51,53].

Cosmetical and pharmaceutical fields frequently use liposomes as nanocarriers but also food industry has increased its interest in using this form of delivery system for food preservation [53,55].

Liposomes have been used as delivery systems for citrus EO compounds such as limonene with applications in the food industry. In 2018, Dhital et al. [59] used the thin-film dehydration method in the fabrication of liposomes loaded with limonene for potential use in shelf-life extension and quality preservation of strawberries. The compound of interest was added under continuous sonication and nanometric liposomes had been formed with the capacity to enhance the shelf-life of strawberries and their post-harvest qualities. D-limonene liposomes were tested by coating strawberries after harvest and depositing them in a sterile environment in order to determine specific markers of shelf-life stability (e.g., total CO_2_ in the deposit room, marker for spoilage microorganism growth, total moisture, pH-changes). Limonene-coated liposomes proved to influence in a positive way the principal factors in shelf-life improvement [59]. D-limonene was also successfully added into liposomal structures by Umagiliyage et al. [60] with the outcome of protecting blueberries after harvest. In this case, the authors compared the untreated berries with ones treated with limonene/water/limonene-loaded liposomes. The results confirmed the assumptions of the in vitro studies on typical spoilage microorganisms, that limonene-loaded liposomes confer significant protection for blueberries against food-borne pathogens [60]. There are also other examples of essential oil compounds (e.g., carvacrol and thymol) loaded into liposomal vesicles with the above-mentioned method [61], or different essential oils such as rosemary or Greek sage [62].

Table 2 presents the average diameter, PDI values, Zeta potential, and time storage for some liposomal nanostructures reported in the literature, loaded with different citrus EOs obtained by different methods.

The thin-layer dispersion method is considered to be a conventional method. It consists of the dissolution of the coating materials into organic solvents, followed by the evaporation and removal of organic solvents under reduced pressure. This process leads to a thin lipid film that is re-dissolved in an aqueous medium when the lipid film can self-assemble into bilayer liposomal vesicles [50].

By the sonication method, essential oils of cinnamon, clove, and lemongrass were entrapped into liposomal vesicles with a size range between 61 and 138 nm depending on the amplitude and the sonication time. All sizes presented a PDI below 0.330 and very good stability with Zeta potentials below −42 mV [63].

Citrus lemon var. pompia EO and its main compound, citral, have been successfully embedded into liposomes in concentrations of 12, 25, and 50 mg/mL with an efficiency of up to 91% for the essential oil and 94% for the main compound. The sizes of the liposomes containing EO ranged from 110 to 117 nm with a PDI index around 0.300 and Zeta potential lower than −85 mV, indicating extremely good stabilities. Citral liposomes had lower diameters from 97 to 105 nm with PDI from 0.350 up to 0.400 and a bit higher Zeta potential of around −70 mV. All these indicators showed effective encapsulation of citrus essential oils with promising results regarding the stability of nanostructures. The antibacterial activity of loaded liposomes against *S. mutans* was assessed, with negative results for the EO and promising results for the citral-loaded liposomes [64]. Additionally, Usach et al. studied the same active compounds for the treatment of skin and mucosal infections. They obtained an average size of the EO EO-loaded liposomes of 152 nm with a PDI of 0.31 and a Zeta potential of −74 mV. The citral-loaded liposomes had an average size of 129 nm with a PDI of 0.32 and a Zeta potential of −72 mV. Both liposomal vesicles showed excellent biocompatibility and promising antibacterial activity against *E. coli*, *S. aureus*, and *C. albicans* [65].

**Table 2:** Comparison of physical-chemical parameters of liposomal nanostructures loaded with different citrus EOs obtained thin-film dehydration and sonication methods.

Active Compound	Preparation Method	Particle Size (nm)	PDI	Zeta Potential (mV)	Storage Time	Reference
D-limonene	Thin-film dehydration	Nanometric scale	-	-	14 days	[59]
		100.2	-	-	63 days	[60]
	Ethanol injection	42	-	-	-	[66]
Citrus limonene v. pompia	Sonication	110–117	0.300	−85	-	[64]
		152	0.31	−74	-	[65]
Citral	Sonication	97–105	0.350–0.400	−70	-	[64]
		129	0.312	−72	-	[65]
Bergamot EO	Thin-film dehydration	185–188	0.230	−2.95	-	[3]
Citronella EO	Sonication	91.5–104.8	0.231–0.277	−38.9–44.8	-	[2]

## 3. Polymer-Based Nanostructures for Citrus Essential Oils

Among all polymers, chitosan is by far the most widely used for nano-encapsulation of citrus EOs. Chitosan has been recognized as an encapsulation material with an affinity for essential oils and natural extracts [67]. Interest in the use of chitosan for nanostructures synthesis has grown in the last decade due to its incontestable properties of chitosan, such as biocompatibility, bioavailability, non-toxicity, and abundance in nature [68], and also due to its ability to enhance antibacterial properties of essential oils [69].

Chitosan is a highly used polysaccharide formed by acetylated (N-acetyl-D-glucosamine) and deacetylated (D-glucosamine) units with a molecular weight ranging from 3.8 to 2000 kDa and a deacetylation percentage between 40 and 98% [70,71]. By depolymerization, low molecular weight chitosan can be obtained (chitooligomers). These oligomers possess superior bioavailability and other appealing properties such as antimicrobial and antifungal [71,72,73].

Chitosan nanostructures (CNSs) are a pressing subject in research fields on the strength of their antioxidant activity. A combination of chitosan and citrus EOs nanostructures leads to a synergetic effect and increase in the antioxidant potential adding important value in food preservation [10] and food packaging [11].

CNSs can be obtained by different methods such as ionic gelation, reverse micelles, emulsification, coacervation, spray-drying, self-assembly, and nanoprecipitation [74]. The most frequently reported methods are ionic gelation and nanoprecipitation, but also nano-emulsification.

Table 3 presents the average diameter, PDI values, Zeta potential, for CNSs loaded with citrus EOs, and obtained by different synthesis methods.

Other polymers were also reported for encapsulation of CEOs, but the particles used in those cases were in the majority of the cases in the micrometer range. Such examples include polyethylene glycol [75,76] and different mixtures of polymers such as alginate, gelatin, Arabic gum, maltodextrin, cellulose [77,78,79], and poly-(methyl methacrylate)-based polymers [80].

Ionic gelation for the formation of chitosan nanostructures (CNSs) has proved to be effective in the encapsulation of different citrus EOs [68,81,82,83]. The ionic gelation method is based on the interaction of opposite-charged macromolecules [74]. The nanoparticle preparation is based on the cross-linking of chitosan and pentasodium triphosphate (TPP) [82]. The gelation process is affected by various factors such as pH, the ratio of ingredients, and the way of mixing, leading to different sizes and stability of nanostructures [74].

As examples, CNSs loaded with *Citrus aurantium* EO were reported by the ionic gelation method. Their ability to enhance the antioxidant activity and control spoilage for *Agaricus bisporus* in cold storage conditions was demonstrated. In co-dependency with the ratio of chitosan and EO, the obtained nanoparticles varied in size from 39 to 47 nm. With a PDI from 0.27 to 0.42 and a Zeta potential between +27 and +50 mV. At a ratio of chitosan:EO of 1:0.75 it was remarked a size of 41.74 nm with a PDI of 0.39 and a Zeta potential of +38 mV [82]. Additionally, using ionic gelation Song et al. obtained chitosan nanostructures loaded with mandarin EO at a chitosan:EO ratio of 1:0.5 with an encapsulation efficiency of 82.35%, stable over time with a Zeta potential of +31.64 mV and an average size of 158.6 nm. In addition, these nanostructures loaded with mandarin EO showed promising results in antibacterial inhibition for *S. aureus* and *E. coli*. In addition, all the developed nanostructures showed antibiofilm properties, which were more pronounced with increasing the EO content. According to the above-mentioned properties, the nanostructures also had promising properties in pork meat preservation [84].

The ionic gelation method by cross-linking of chitosan with TPP is a widely used method for chitosan loaded with citrus EO nanostructures. The method was used not only in the food industry but also in leather production. With this purpose Velmurugan et al. obtained chitosan nanostructures loaded with orange EO with an encapsulation efficiency of 40.23%, size of 213.6 nm, and antibacterial properties against *B. cereus*, *B. subtilis*, *A. fumigatus* and *M. phaseolina* [85].

CNSs can also be formed by using the freeze-drying technique but also with ionic gelation as the nano-encapsulation process. Hasani et al. reported the synthesis of nanocapsules based on chitosan and modified starch (HICAP) with lemon EO as cargo. The motivation for these two encapsulation ingredients stands in their ability to complex ionically at food pH and confer stability and protection for the cargo. The encapsulation efficiency for a ratio of chitosan: HICAP 1.5:8.5 was 85.437%, with an average size of 339 nm, Zeta potential of +44.233 mV, and PDI of 0.424 [67].

In addition to citrus EO, the ionic gelation technique was also used to obtain various chitosan-loaded nanostructures with Lemongrass EO [86], tarragon EO [87], *Piper nigrum* EO [88] carvacrol [83], clove EO [68,89], oregano EO [90], *Zataria multiflora EO* [91], etc.

Another widely used method, nanoprecipitation, implies the use of two miscible phases called solvent and non-solvent. The polymer (chitosan in the present case) and the active ingredient (the citrus EO) ideally solve in the solvent phase and have no dissolution properties for the non-solvent phase. This method is dependent on the phenomena accompanying the solubility properties. The solvent has to be a semi-polar substance with high solubility properties for the polymer used (e.g., chitosan) and the active ingredient (e.g., citrus EO). In the solvent phase, surfactants can also be added in order to improve the stability of the final nanostructures. The principle of the method consists of the dissolution of the polymer and active ingredient in the solvent phase (e.g., ethanol) which is afterward added slowly into the non-solvent phase (e.g., water). As mentioned, the non-solvent phase ideally has no dissolution properties for the active ingredient and the polymer, and in contact with the two solvents, the polymer suffers a supersaturation phenomenon that is essential in the nanostructure formation. Nanoprecipitation is divided into four important stages called supersaturation, nucleation, growth, and coagulation. Depending on the shearing speed of the mixture, these four phenomena can lead to different dimensions of the capsules, and also to different kinetic stability of the suspension [92].

Sotelo-Boyá et al. compared two techniques based on CNSs loaded with lime EO. The nanoprecipitation technique used a 0.05% *w*/*v* chitosan in acetic acid solution combined with a 20% essential oil and methanol solution under moderate magnetic stirring for the precipitation phase and in the end, the solvents were evaporated using a rotary evaporator. For this method nanoparticles with sizes between 25 and 50 nm were obtained with poor stability and a Zeta potential of +10 mV. Another method was nano-encapsulation. The method assumed the incorporation of a solution composed of lecithin, ethanol, essential oil, and acetone into a 0.5 *w*/*v* chitosan. The solvent evaporation was achieved at a maximum temperature of 40 °C. The size of nanoparticles obtained ranged from 190 to 700 nm with good stability in time and a Zeta potential of +57.0 mV. For both methods, an antibacterial study for the nanostructures loaded with lime EO was conducted with increased inhibition zones for *S. aureus* (3.3 cm vs. 1.2 cm), *L. monocytogenes* (1.8 vs. 1.3 cm), *S. dysenteriae* (3.5 cm vs. 1.5 cm) and *E. coli* (3.0 cm vs. 1.0 cm) for the ones obtained by the nanoprecipitation method [93].

Another polymer that was reported for the synthesis by nanoprecipitation of polymer nanoparticles based on bergamot and orange EOs is Eudragit RS100, which is a cationic copolymer of ethyl methacrylate, methyl methacrylate, and methacrylic acid esterified with quaternary ammonium groups [80]. The prepared nanostructures had mean diameters of 27–200 nm and very good stability, as indicated by the positive Zeta potential values of +39 and +74 mV. The Eudragit EO particles had very good antimicrobial properties against *E. coli* (90% inhibition compared to around 50% inhibition provided by the non-loaded particles) and they were also tested for their potential to enhance the shelf life of freshly squeezed, non-sterilized orange juice. Good antimicrobial properties also against *E. coli* from the orange juice were reported, with 90% bacterial inhibition after 24 h and 80% after 1 week of storage at 25 °C.

Nano-emulsification is another suitable method for developing nanostructures based on polymers and EOs. The EPI method, already described, was used to form chitosan-based nano-emulsions of orange EO [47]. The droplet size was below 60 nm the stability over time was larger than 3 months at 2–4 °C. Antibacterial activity against *E. coli* was reported in vitro, as well as in apple and orange juices (alone or synergistic).

Even though lipid-based and polymer-based nanostructures are the most widely used for encapsulation of citrus EOs, polysaccharides and proteins were also reported for such purposes [94].

## 4. Conclusions

There is a real need for developing nanosystems for promoting the delivery of citrus EOs and for enhancing their stability. Citrus EOs have promising properties, but large-scale applicability is limited by their instability. Several strategies and methodologies have been proposed for the encapsulation of citrus EOs, the most common being the lipid-based and polymer-based approaches, as described in the current review. From the results reported so far, and from the formulations that were developed, some also have promising properties that would make them suitable for the food sector, such as antibacterial and antioxidant. Still, there is a need for research in this direction.

## Figures and Tables

**Table 1:** Comparison of physical–chemical properties for NEs produced with citrus essential oils and different types of emulsifiers.

EO Type	Technique	Emulsifier	Particle Size– Average (nm)	PDI	Storage Time	Reference
Orange EO	Spontaneous emulsification and ultrasonic emulsification	Tween 80	68.3	0.4–0.2	35 days	[46]
	Phase inversion					
	Ultrasonic homogenization	Tween 80	94.66	0.22	2 weeks	[48]
	Ultrasonic homogenization	Tween 80	94.66	-	2 weeks	[38]
	Ultrasonic homogenization	Tween 80	21.75	0.753	12 weeks	[37]
D-limonene	High-pressure homogenization	Soy lecithin	235.9	-	4 weeks	[35]
	High-pressure homogenization	Tween 20 + Glycerol monooleate	130.9	-		[35]
	High-pressure homogenization	Clear gum	365.7	-	4 weeks	[35]
	High-pressure homogenization	Soy lecithin	239	0.18		[36]
	High-pressure homogenization	Pea proteins	184	0.14		[36]
	High-pressure homogenization	Sugar ester	169	0.07		[36]
	High-pressure homogenization	Tween 20 + Glycerol monooleate	228	0.09		[36]
	Phase inversion	Polyoxyethylene 40	112			[45]
Mandarin EO	Ultrasonic homogenization	Tween 80	79.14	0.442	2 weeks	[38,48]
	High-pressure microfluidization	Tween 80	17	-	8 weeks	[40]
Grapefruit EO	Ultrasonic homogenization	Tween 80	81.05	0.465	2 weeks	[38,48]
Lemon EO	Ultrasonic homogenization	Tween 80	47.40	0.413	2 weeks	[38,48]
	Ultrasonic homogenization	Tween 80	30.2	0.686	1 week	[49]
	Ultrasonic homogenization	Tween 80	181.5	0.114		[39]
Lime EO	Phase inversion	Tween 80	21	0.444	4 weeks	[43]
Bergamot EO	High-pressure homogenization	Glycerol monooleate: Tween 20 (1:1)	138	0.14	8 weeks	[14]

**Table 3:** Comparison of physical-chemical parameters of chitosan nanostructures of different citrus EOs obtained ionic gelation, nanoprecipitation, nano-encapsulation, and nano-emulsification methods.

EO Type	Preparation Technique	Particle Size (nm)	PDI	Zeta Potential (mV)	Reference
Orange EO	Ionic gelation	39–47	0.39	38	[82]
	Ionic gelation	213.6	0.69	-	[85]
	Nanoemulsification (EPI)	58.2	0.262	-	[47]
Lime EO	Nanoprecipitation	25–50	-	10	[93]
	Nano-encapsulation	190–700	-	57	[93]
Mandarin EO	Ionic gelation	158.6	-	31.64	[84]
Lemon EO	Ionic gelation	339.333	0.424	44.233	[67]

## Data Availability

Not applicable.
